# Versatile construction of van der Waals heterostructures using a dual-function polymeric film

**DOI:** 10.1038/s41467-020-16817-1

**Published:** 2020-06-15

**Authors:** Zhujun Huang, Abdullah Alharbi, William Mayer, Edoardo Cuniberto, Takashi Taniguchi, Kenji Watanabe, Javad Shabani, Davood Shahrjerdi

**Affiliations:** 1 Electrical and Computer Engineering, New York University, Brooklyn, NY 11201 USA; 2 King Abdulaziz City for Science and Technology, Riyadh, 11442 Saudi Arabia; 3 Center for Quantum Phenomena, Physics Department, New York University, New York, NY 10003 USA; 4 National Institute of Materials Science, 1-1 Namiki Tsukuba, Ibaraki, 305-0044 Japan

**Keywords:** Two-dimensional materials, Electronic properties and devices, Synthesis and processing

## Abstract

The proliferation of van der Waals (vdW) heterostructures formed by stacking layered materials can accelerate scientific and technological advances. Here, we report a strategy for constructing vdW heterostructures through the interface engineering of the exfoliation substrate using a sub-5 nm polymeric film. Our construction method has two main features that distinguish it from existing techniques. First is the consistency of its exfoliation process in increasing the yield and in producing large (>10,000 μm^2^) monolayer graphene. Second is the applicability of its layer transfer process to different layered materials without requiring a specialized stamp—a feature useful for generalizing the assembly process. We demonstrate vdW graphene devices with peak carrier mobility of 200,000 and 800,000 cm^2^ V^−1^ s^−1^ at room temperature and 9 K, respectively. The simplicity of our construction method and its versatility to different layered materials may open doors for automating the fabrication process of vdW heterostructures.

## Abstract (web summary)

Heterostructure stacking of 2D materials is crucial for fundamental studies and device applications. Here, the authors report heterostructures based on exfoliated flakes of graphene with large lateral area sizes and record high mobility of 200,000 cm2/Vs at room temperature.

## Introduction

Van der Waals (vdW) heterostructures are an exciting playground for scientific discoveries among different disciplines^1–11^. These structures are made by stacking different layered materials that are held together by vdW forces. The vast diversity of layered materials can result in a large number of possible combinations of layered material stacks, which could be employed for future research in a wide range of fields, from many-body physics to electrochemistry. However, efficient methodologies for constructing diverse vdW heterostructures are still lacking. What is needed is a versatile construction method with the ability to build arbitrary stacks of high-quality layered materials using an identical fabrication process.

**Fig. 1:**
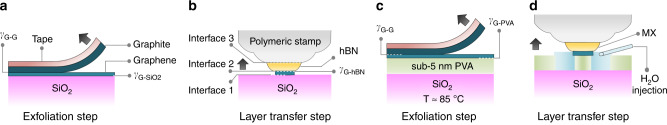
Fabrication of vdW heterostructures. Schematic representations of **a** exfoliation and **b** layer transfer processing steps in the conventional fabrication methods. In those methods, *γ*_G-SiO2_ must exceed *γ*_G-G_ during exfoliation. Moreover, *γ*_G-hBN_ must exceed *γ*_G-SiO2_ during layer transfer. *γ* denotes the adhesion energy at the interface between two respective materials in contact with each other. These requirements are conflicting, thus complicating the process of heterostructure fabrication. We introduce a simple interface engineering method to overcome this dilemma by using a nanoscale PVA release layer. **c** In our process, PVA can be tuned to promote exfoliation. **d** Moreover, selective removal of PVA enables a high-yield layer transfer of the exfoliated flakes without requiring a specialized stamp, hence relaxing the choice of the stamp material (i.e., MX). The black arrows indicate the direction of the applied displacement force during the exfoliation and layer transfer steps.

Continuous progress on developing standard processes for assembling exfoliated flakes has made vdW heterostructures more accessible to the research community^12–18^. Past research has developed those standard processes around the direct exfoliation of layered materials on SiO_2_ substrates^19^. This method has remained popular because of the ability to produce clean (i.e., homogeneous and low-disorder) layered materials. Figure 1a, b show the schematic representation of this conventional method of direct exfoliation on SiO_2_ and the following layer transfer of the exfoliated flake (e.g., graphene) onto a stamp. However, despite this significant progress, efficient and high-yield fabrication of diverse heterostructures remains a difficult task.

A major barrier to the efficient construction of vdW heterostructures using the conventional method is the opposing requirements of the exfoliation and layer transfer steps. In principle, promoting the adhesion between the substrate and the layered material improves the exfoliation process^20,21^. However, the adhesion at the flake-substrate interface must be ideally weak during the transfer process for two important reasons. First, it results in high transfer yields by facilitating the release of the exfoliated flake. Second, it relaxes the requirement on the adhesion strength of other interfaces within the stamp (e.g., interface 2 and 3 in Fig. 1b), since the adhesion in those interfaces must exceed that of the flake-substrate interface.

Because of these opposing requirements, the conventional exfoliation method suffers from small flake size, a low number of flakes, and inconsistency of the exfoliation outcome among different trials^20,21^. Hence, the exfoliation step is typically repeated until there are enough layered material flakes available for constructing the heterostructure. Further, the opposing requirements of the adhesion strength critically limit the choice of the transfer stamp for detaching the layered material flake from SiO_2_. The tear-and-stack method using a hexagonal boron nitride (hBN) flake (see Fig. 1b) is currently the most common method for building hBN-encapsulated heterostructures^12,16,22^. However, to build arbitrary stacks of layered materials, each research lab has developed ad hoc solutions^6,23–25^. The above limitations make the fabrication of vdW heterostructures using existing methods non-standard, and tedious.

An efficient and high-yield fabrication process could be achieved if the adhesion at the flake-substrate interface can be optimized independently during the exfoliation and transfer steps. Here, we report a strategy that achieves this objective. Specifically, we modify the SiO_2_ substrate by coating it with a sub-5 nm poly(vinyl alcohol) (PVA) film (see Fig. 1c). We find that the adhesion strength of the PVA film can be tuned to promote the exfoliation of layered materials. For the layer transfer process, we remove the PVA film on-demand, freeing the exfoliated flakes from the substrate (Fig. 1d). Our construction method is simple, high-yield, and generalizable to different layered materials.

## Results

### Consistent exfoliation and layer transfer via interface engineering

The exfoliation process begins by coating the SiO_2_ substrate with a sub-5 nm PVA film (see Methods). A Scotch tape containing graphite crystals is then pressed onto the substrate, followed by a brief heat treatment at *T* ≈ 85 °C (which is close to the glass transition of PVA). The tape is then pulled back slowly. We found that the heat treatment step noticeably promotes the exfoliation of graphene, resulting in large flakes, a high number of flakes per sample, and consistency among different exfoliation runs (discussed later). The illustration in Fig. 1c shows the PVA-assisted graphene exfoliation (PAGE) process.

**Fig. 2:**
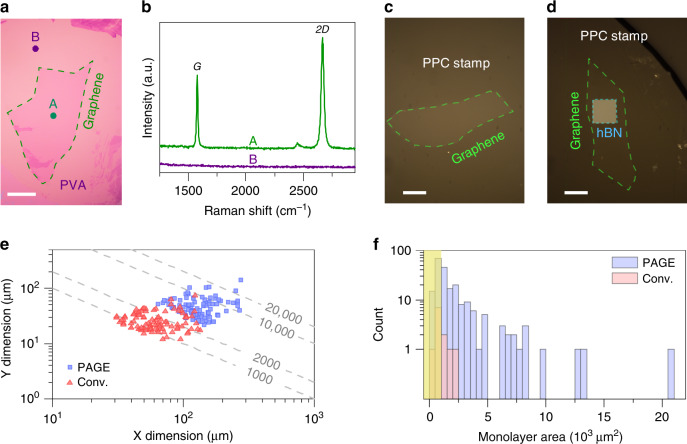
PVA-assisted graphene exfoliation (PAGE). Our method allows the use of existing inspection techniques using **a** optical and **b** Raman microscopes for identifying monolayer flakes. Scale bar in **a** is 50 μm. The Raman spectra in the **b** correspond to the regions in the optical image of the **a** marked with “A” and “B”. We showed the effectiveness of our layer transfer method by picking up monolayer graphene using **c** a PPC stamp, and **d** an hBN/PPC stamp. Scale bars are 20 μm. **e** PAGE reliably produced very large monolayer graphene flakes, which were not attainable by the conventional method in our experiments. The dashed lines are guides to the eye and represent the product of X and Y dimensions equal to 1000, 2000, 10,000, and 20,000 μm^2^. **f** The histogram plot showing the distribution of the monolayer flake area for the samples obtained from 15 consecutive exfoliation trials. The data indicate that PAGE is 20 times more likely than the conventional method (denoted as Conv.) to produce monolayer graphene with an area larger than 1000 μm^2^ (notice the distribution beyond the yellow shading). The yellow shading denotes flakes with an area of <1000 μm^2^.

Identifying the number of layers within an exfoliated graphene flake on a PVA-coated SiO_2_ can be done with well-established practices^19,26,27^. We first identified candidate flakes through optical inspection. Figure 2a shows an optical image of a 10,000 μm^2^ monolayer graphene produced using PAGE, illustrating the excellent optical contrast between graphene and PVA. Subsequent Raman measurements on the candidate flakes are essential for obtaining the exact number of layers within each flake. We observed that the PVA film does not interfere with the Raman signature of graphene (Fig. 2b). This property would allow the use of established Raman techniques (e.g., see refs. ^27–29^) for studying the structural properties of the exfoliated graphene flakes on PVA.

Our simple interface engineering method is effective in producing large monolayer flakes (see Supplementary Note 1 and Supplementary Fig. 1). Figure 2e compares the dimensions of the 100 largest PAGE flakes against those produced using the conventional method. We obtained these flakes from 33 PAGE samples and 77 conventional exfoliation samples. Each exfoliation trial comprised one sample and the size of each sample was 1 × 1 cm^2^. The comparison between these two methods shows the ability of our method to produce large monolayer graphene flakes. The difference in the number of samples between the two methods in this analysis is because PAGE consistently yields a higher number of flakes per sample than the conventional method. To study the yield and the consistency of the exfoliation, we analyzed the monolayer flake area distribution on samples from 15 consecutive exfoliation trials, which we performed for both methods. We accounted for all samples, including those with no monolayer flakes. The results in Fig. 2f indicate that, compared to the conventional method, our method is about 20 times more likely to produce monolayer flakes that are bigger than 1000 μm^2^ in each sample.

Figure 1d shows the schematic illustration of our layer transfer method. The process begins with landing the transfer stamp onto the exfoliated flake. A drop of water is then applied locally near one end of the flake. The stamp is withdrawn gradually at a slow speed of 20–30 μm s^−1^ until the flake is fully released (Supplementary Movie 1). The slow speed ensures that the stamp remains in full contact with the flake while allowing the water to permeate underneath the flake. Depending on the size of the flake, the release process takes only a few to ten seconds. Because of the local application of the water drop in our process, the exfoliated flakes in other regions of the substrate remain intact and usable for future fabrication experiments (Supplementary Fig. 4).

Our layer transfer method relaxes the adhesion requirements of the stamp material. This is a major advance for realizing arbitrary stacks of layered materials. We demonstrated this capability, discussed next, by transferring exfoliated monolayer graphene flakes onto two stamps that are known to have vastly different adhesion strength to graphene.

The first stamp consisted of a poly-propylene carbonate (PPC) coating on a polydimethylsiloxane (PDMS) layer, commonly used in the stacking process of layered materials^12,15^. The adhesion strength of this stamp is insufficient for detaching exfoliated graphene flakes produced by the conventional method. In our layer transfer method, however, this polymeric stamp can successfully pick up large monolayer graphene flakes from the exfoliation substrate, as shown in Fig. 2c.

We also produced a different stamp by attaching an hBN flake onto the PPC/PDMS stamp^12,15,16,18^. Figure 2d shows the optical image of a large monolayer graphene flake, which was transferred fully from the substrate onto the stamp using our layer transfer method. In this image, notice the graphene layer in regions outside the rectangular hBN flake, indicating that the layer transfer is independent of the adhesion to hBN. This outcome of our layer transfer process contrasts with the tear-and-stack method using a PPC stamp^12,15,16^, where the shape of the transferred flake conforms to that of the hBN flake. Fabrication of a similar stack structure using the conventional method is possible only by changing the polymer coating of the stamp (for example, see ref. ^14^).

### Fabrication and material characterization of graphene heterostructures

Next, we used monolayer graphene transferred onto the two different stamp types (i.e., PPC and hBN/PPC) for building two groups of heterostructures: graphene-on-hBN (GB) and graphene encapsulated in hBN (BGB) stacks. To do so, we first assembled these stacks on the stamp itself by picking up the bottom hBN flake from SiO_2_. The full stacks were then laminated at temperatures below 110 °C onto the support substrates. We performed the lamination following a similar procedure in ref. ^15^. Prior to our study, the construction of these two heterostructures involved developing different strategies and processing steps (e.g., see refs. ^1,12,14,23,30^). The versatility of our method overcomes this technical difficulty for fabricating diverse layered material stacks.

Direct exfoliation of layered materials on clean SiO_2_ substrates mitigates the chance of material degradation due to contaminants, enabling the fabrication of heterostructure devices with high carrier mobility^12^. In our method, the layered material comes into contact with PVA and water, raising a valid concern about the degradation of graphene quality and its suitability for making high-mobility devices. To understand the effect of graphene exposure to PVA and water, we studied the surface properties of graphene using high-resolution atomic force microscopy (AFM). Our AFM measurements indicate an increase of both the mean phase and the average roughness of graphene after exposure to PVA and water (see Supplementary Note 11, Supplementary Figs. 11–20, and Supplementary Table 3). While not possible to separate the effects of PVA and water, these experiments suggest an apparent negative impact on the graphene surface properties. However, as we discuss next, this is not an impediment for producing graphene heterostructures with clean interfaces.

**Fig. 3:**
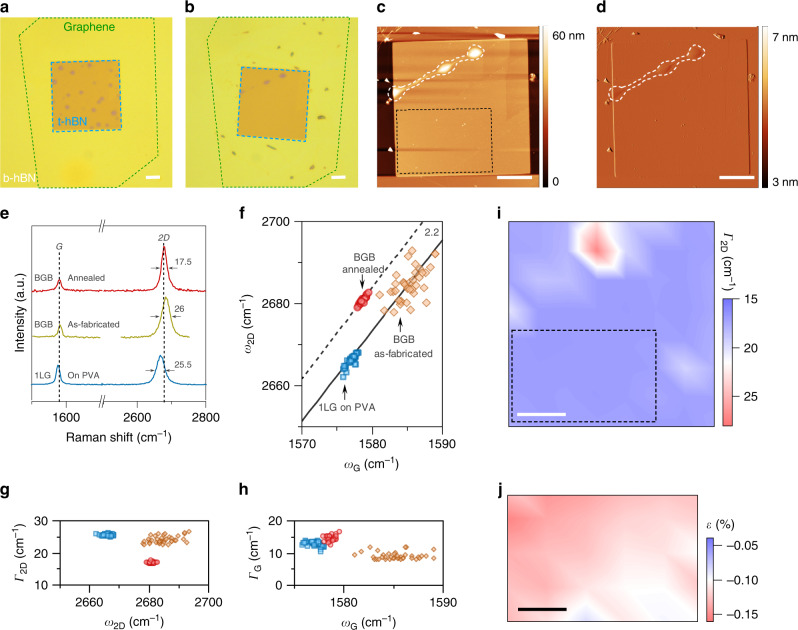
Evaluation of graphene material quality. Optical images of a BGB stack **a** after lamination (i.e., as-fabricated) and **b** after UHV annealing, indicating the effectiveness of the annealing step in removing blisters. High-resolution **c** AFM topography and **d** AFM topography error images confirm the cleanliness of the annealed BGB stack. Notice the remaining blisters in the top left corner of the AFM images. The black dashed box represents the region, which we used for comparing the Raman data before and after UHV annealing. **e** Typical Raman spectra of monolayer graphene (1LG) obtained at the different stages of the BGB stack fabrication. The black dashed lines mark the position of G and 2D lines after UHV annealing. The numbers next to the 2D lines give the FWHM. **f** The plot of *ω*_2D_ against *ω*_G_. The dashed and solid black lines have a slope of 2.2. The scatter plots of (**g**) Γ_2D_ against *ω*_2D_, and **h** Γ_G_ against *ω*_G_. The symbols in these two plots correspond to the data in the **f**. **i** The spatial map of Γ_2D_ of the annealed BGB stack, confirming the ability of Raman to provide precise information about the location of blisters. The black dashed box in this plot is the same region as in the AFM image of the **c**. **j** The estimated strain amplitude in the marked rectangular region. All scale bars are 5 μm.

Atomically clean interfaces are required for superior carrier transport in graphene heterostructures. Therefore, we examined the structural properties of our BGB stacks using AFM and Raman spectroscopy. In Fig. 3a, we show the optical image of a BGB stack after lamination. The data clearly shows that the as-fabricated BGB heterostructures made using our PVA-based approach suffer from microscopic blisters. While the exposure to PVA and water could amplify the likelihood of blister formation, we attribute this issue primarily to the sub optimal conditions of our lamination process (see Supplementary Note 12 for details). Inspired by previous reports that the high-temperature processing can mobilize blisters^6,15,18^, we performed annealing on fully fabricated BGB stacks in ultra-high vacuum (UHV) at 400 °C (see “Methods”). Post-fabrication annealing at similar temperatures is routinely performed for cleaning fully formed heterostructures made of flakes prepared by the conventional exfoliation (e.g., see refs. ^6,9,31–34^). Interestingly, we observed that this annealing step is highly effective in removing blisters. In Fig. 3b–d, we show the optical and AFM images of the BGB stack of Fig. 3a after the UHV annealing step. The data illustrate that a large portion of the BGB stack is blister free, indicating the effectiveness of the UHV anneal in cleaning the graphene interfaces with the top and bottom hBN (t-hBN and b-hBN) flakes.

Next, we used Raman spectroscopy to investigate further the material properties of graphene at three different stages of the BGB stack fabrication process. Figure 3e shows the typical Raman spectrum of graphene after its exfoliation on PVA, after the lamination of the BGB stack, and after the UHV annealing step. The visible differences in the characteristics of the G and 2D peaks of these three spectra indicate detectable changes in the graphene properties during the stack fabrication, which we elaborate below.

The data in Fig. 3e show a noticeable blueshift of the 2D line after encapsulating graphene in hBN. This observation has been attributed to the effect of dielectric screening by hBN on the electronic structure of graphene^35^. We also observed a marked reduction in the full-width at half-maximum (FWHM) of the 2D line (Γ_2D_) after the UHV annealing of the BGB stack. We attribute this observation to the removal of blisters. In particular, previous studies reported that Γ_2D_ is sensitive to structural deformation of graphene, for example, those caused by blisters in a BGB stack^18,36^. It has been observed that its value is generally >20 cm^−1^ in blistered regions of a BGB stack and is <20 cm^−1^ in blister-free regions. Taking advantage of this knowledge, we observed that the spatially resolved measurement of Γ_2D_ on the annealed stack, shown in Fig. 3i, provides precise information about the location of blisters, consistent with the AFM images in Fig. 3c, d. The 2D lines recorded in the blister-free region of the stack, marked with a dashed box, have a Γ_2D_ of 17 ± 0.45 cm^−1^. In contrast, the Γ_2D_ in the same region before annealing is above 22 cm^−1^, comparable to those of exfoliated graphene on PVA (see Fig. 3g). The marked decrease of Γ_2D_ in this region of the sample after the UHV anneal is an indication of the reduction in the nanometer-scale strain inhomogeneities^36^.

Analyzing the spatial Raman map in this region of the sample also revealed the broadening of the G lines (i.e., Γ_G_) after the UHV annealing (Fig. 3h). This observation suggests the reduced charge carrier doping of graphene after the UHV annealing^36–38^. Another important observation from this plot is the reduced variations of *ω*_G_ (i.e., Δ*ω*_G_) after the UHV annealing. To examine the origin of this observation, we plotted *ω*_2D_ against *ω*_G_ (Fig. 3f). The data in this plot provide critical information on how graphene properties change at different stages of our BGB fabrication process. In particular, the data points of both the graphene on PVA and the blister-free region of the annealed stack scatter tightly along a line with a slope of 2.2 (the solid and dashed black lines in the plot). This indicates that Δ*ω*_G_ at these two stages is mostly due to the strain variations (Δ*ε*) and that the contribution to Δ*ω*_G_ due to doping variations is negligible^18,36,39^. In contrast, the noticeable spread of the data points for the as-fabricated stack suggests significant variations of both doping and strain within the sample before the UHV annealing.

Another valuable information obtainable from this plot is the estimation of strain variations in graphene. In particular, local strain fluctuations can critically limit carrier transport in graphene devices^40^. Hence, we quantified the strain variations in the blister-free region of the BGB stack, which we utilize for device fabrication. The estimated amplitude of strain using Raman depends on the uniaxial or biaxial nature of the strain in graphene^41–43^. However, the Raman spectrum of graphene at low strain gives no discernible signature for differentiating the nature of the strain^41,43^. In our calculations, though, we assumed a uniaxial strain because it gives a worst-case estimate for the variations of the strain amplitude in graphene (i.e., about three times larger than biaxial)^41–43^. In Fig. 3j, we show the spatially resolved distribution of the calculated strain amplitude in the blister-free region of the UHV-annealed BGB stack. The data indicate the presence of tensile strain in graphene with an average amplitude of ≈0.123%. This level of strain is low, and its effect on the electronic band structure of graphene is negligible^44^. More importantly, the small spread in *ω*_G_ translates into Δ*ε* ≈ 0.067%. Moreover, the shift of data points towards lower values of *ω*_G_ after UHV annealing can be interpreted as a reduction in the charge carrier doping of graphene^37^. This observation is consistent with the broadening of the G lines after UHV annealing, discussed earlier in Fig. 3h.

Our material characterization study using Raman spectroscopy and AFM imaging provides strong evidence that our technique is capable of producing high-quality graphene heterostructures. In particular, our BGB stacks have desirable properties (e.g., low-residual doping and negligible strain variations), making them attractive for high-performance device applications.

### Transport studies of graphene heterostructure devices

**Fig. 4:**
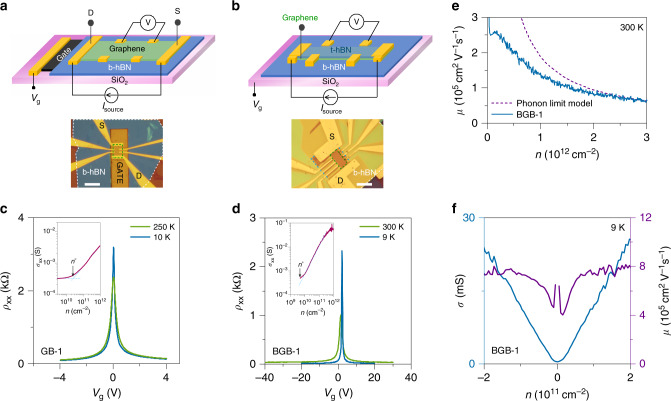
Transport characteristics of graphene heterostructures. We fabricated gated-Hall bar devices from two types of graphene heterostructures: **a** graphene-on-hBN (GB) and **b** graphene encapsulated in hBN (BGB). The dashed green box in the optical images shows the device active region. The scale bars are 10 μm. The narrow FWHM of the four-point longitudinal resistivity (*ρ*_xx_) of the **c** GB and **d** BGB devices suggests a low-charge inhomogeneity (n^*^) in graphene. The insets in **c** and **d** show the estimated n^*^ of the GB and BGB devices. **e** The carrier-density-dependent mobility of the BGB-1 device at room temperature, giving mobility in excess of 200,000 cm^2^ V^−1^ s^−1^ at low carrier densities. For comparison, we provided the theoretically predicted acoustic phonon-limited mobility^45^. **f** The corresponding low-temperature mobility of the BGB-1 device is 800,000 cm^2^ V^−1^ s^−1^ at low carrier densities.

We fabricated gated-Hall bar devices from the GB and BGB stacks to evaluate their electronic properties. To build these structures, we applied our exfoliation and layer transfer methods only to graphene and used the conventional method for producing hBN flakes. This experimental design allows us to focus on the effect of our process on graphene quality. Figure 4a, b show the schematic illustrations and example optical images of these gated-Hall bar devices. We used standard nanofabrication processes for building these devices (see “Methods”).

Electrical measurements on our GB and BGB devices indicate the high material quality of monolayer graphene produced by PAGE. Figure 4c shows the typical four-point longitudinal resistivity (*ρ*_xx_) of monolayer graphene in a GB device measured as a function of the back-gate bias (*V*_g_) (see “Methods”). For evaluating the carrier mobility (*μ*), we assumed that a combination of short- and long-range scattering mechanisms controls the carrier transport in graphene^45,46^. As a result, the resistivity of graphene at charge carrier densities far away from the charge neutrality point (CNP) follows *ρ*_xx_ = (e*nμ*_L_)^−1^ + *ρ*_s_, where *n* is the gate-voltage induced carrier density, *μ*_L_ is the mobility due to long-range Coulomb scattering, and *ρ*_s_ is a term due to short-range scattering. At sufficiently low carrier densities but far away from CNP the contribution of *ρ*_s_ to the resistivity is generally negligible, hence *μ* ≈ *μ*_L_^47^. This approach gave *μ* ≈ 47,000 cm^2^ V^−1^ s^−1^ at 10 K for the GB device in Fig. 4c.

While the estimated *μ* of the GB device is comparable with the previously reported counterparts^23,48–50^, it is significantly lower (by >20 times) than the state-of-the-art fully encapsulated graphene devices (for example, see refs. ^12,18^). We attribute the degradation of the carrier mobility primarily to the graphene contamination due to exposure to polymers and solvents. In this device structure, graphene comes in contact with polymers and solvents during both the GB stack construction and the device fabrication. It is, hence, difficult to unambiguously identify the independent contribution of the stack and device fabrication processes to the mobility degradation in this device structure. Unlike the GB structure, however, the top hBN layer in the BGB structure protects graphene from exposure to contaminants during the device fabrication process. Hence, the electrical characteristics of the BGB device provide a direct measure of the graphene cleanliness prepared using our PVA-based technique.

Figure 4d shows the *ρ*_xx_ of a BGB device at room temperature and 9 K (the base temperature of our measurement system). The sheet resistivity of this device (BGB-1) at room temperature is <40 Ω at *n* ≈ 2 × 10^12^ cm^−2^ (Supplementary Note 3 and Supplementary Fig. 3), evidence of high graphene quality. To gain better insight, we then calculated the carrier-dependent mobility of the BGB-1 device at room temperature (Fig. 4e) using the Drude model of conductivity, *σ* = e*nμ*. The carrier density was calculated by considering both the quantum capacitance and the oxide capacitance. For calculations of the oxide capacitance, we used a dielectric constant of 3 for hBN (extracted from the magneto-transport measurements, see Supplementary Note 9 and Supplementary Fig. 9) and measured the thickness of hBN using AFM. The data indicate room-temperature mobility over 200,000 cm^2^ V^−1 ^s^−1^ at low carrier densities. At larger carrier densities (*n* ≈ 3 × 10^12^ cm^−2^) the mobility is ≈62,000 cm^2 ^V^−1 ^s^−1^, which is comparable to the theoretical acoustic phonon-limited mobility calculated assuming a deformation-potential coupling constant (*D*) of 14 eV (Supplementary Note 4)^51^. The theoretical carrier-density-dependent mobility curve in this plot corresponds to a theoretically predicted *ρ*_e-ph_ of ≈32 Ω^52^. We note that the room-temperature carrier mobility of the BGB-1 device improved after a thermal cycle in the measurement system (see Supplementary Note 7, Supplementary Fig. 7, and Supplementary Table 2). Thermal cycling has been previously observed to improve the mobility of BGB devices by modulating the charge inhomogeneity^33^.

We also measured *μ* of the BGB-1 device at 9 K. Using the Drude model of conductivity, we calculated a carrier mobility of ≈800,000 cm^2 ^V^−1^ s^−1^ for this device, as shown in Fig. 4f. The transport characteristics of our BGB device are well within the range of the typical carrier mobility for the state-of-the-art fully encapsulated graphene devices prepared using the polymer-free layer assembly method (for example, see refs. ^12,18^). The electrical characteristics of the BGB devices confirm the ability of our PVA-based technique to produce high-quality graphene devices.

We studied the charge carrier inhomogeneity (n^*^) as a measure of graphene quality. Interactions of graphene with its environment (e.g., variations of chemical doping, charged impurities) can cause spatial charge inhomogeneity close to the Dirac point due to electron-hole (e-h) puddle formation^53,54^. The charge carrier inhomogeneity can be estimated from the gate-induced carrier density below which the conductivity *σ* = (*ρ*_xx_)^−1^ remains unchanged with gating. From the data in the insets in Fig. 4c, d, we found n^*^ to be ≈2 × 10^10^ and <5 × 10^9^ cm^−2^ for the GB-1 and BGB-1 devices. These values represent an upper-bound estimate of the charge inhomogeneity in our graphene samples^40,55^. Further, the monolayer graphene in these devices is nearly intrinsic (i.e., low doping), evident from the peak resistivity position at nearly zero gate-voltage (see *ρ*_xx_ plots in Fig. 4). Measurements on additional GB and BGB devices resulted in similar values for *μ* and n^*^ (see Supplementary Note 6, Supplementary Figs. 5 and 6, and Supplementary Table 1). These observations further confirm that the exfoliated graphene prepared by PAGE is of high quality.

Next, we performed magneto-transport measurements on the BGB-1 device at the base temperature of 9 K. The rationale behind these measurements was to glean further information about the carrier transport properties of the BGB device. In particular, the transport scattering time, which determines carrier mobility, is insensitive to both small- and large-angle scatterings^56^. For this reason, the measurement of mobility alone is insufficient to provide a complete picture of carrier scattering in graphene. In contrast, the elastic scattering time in graphene, which determines the quantum (i.e., Landau) level broadening, is sensitive to small-angle scattering events. Therefore, the outcome of these measurements provides additional information about the graphene quality, which is inaccessible to transport mobility measurements.

**Fig. 5:**
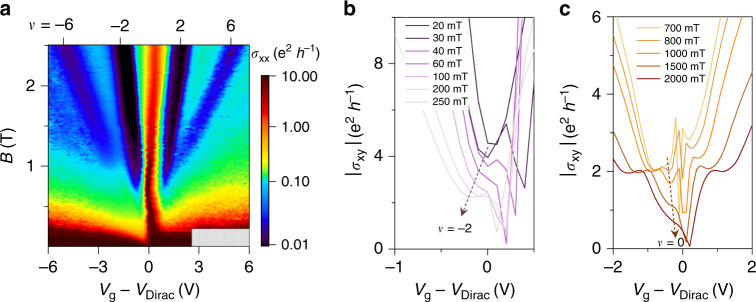
Magneto-transport measurements of graphene. **a** The fan diagram of the longitudinal conductivity (*σ*_xx_) shows well-resolved quantum Hall states at the base temperature of 9 K. The measurements were made on the BGB-1 device of Fig. 4. **b** The transition of Hall conductivity (*σ*_xy_) to *ν* = −2 commences at ≈40 mT. **c** The lifting of the zero-energy Landau level degeneracy begins at below 1 T. These observations indicate the high quality of the two-dimensional electronic system in our graphene heterostructure.

In Fig. 5a, we show the Landau fan diagram of the longitudinal conductivity (*σ*_xx_) for the BGB-1 device. To plot the intensity map of the longitudinal conductivity, we calculated *σ*_xx_ from *ρ*_xx_/(*ρ*_xx_^2^ + *ρ*_xy_^2^), where *ρ*_xy_ is the transverse resistivity. The Landau fan diagram shows well-resolved quantum Hall states (QHS), indicating the high quality of the two-dimensional electronic system in monolayer graphene.

In a two-dimensional electronic system, the quantization begins at a sufficiently strong magnetic field, which yields cyclotron energy larger than the Landau level broadening^57^. Quantifying the elastic scattering time in samples with charge inhomogeneity is highly involved (e.g., see refs. ^58,59^). However, the onset of Shubnikov de Haas can be used for a qualitative assessment of this scattering time by providing an estimate for Dingle mobility at low carrier densities^47^. To do so, we examined the transition of Hall conductivity (*σ*_xy_) to |*ν* | = 2 phase, as shown in Fig. 5b, because this filling factor appears first in the QHS sequence in graphene^60^. The data indicate that the transition commences in fields around 40 mT, corresponding to an estimated Dingle mobility of at least 250,000 cm^2 ^V^−1^ s^−1^ at 9 K. We also observed that the degeneracy lifting of the Landau levels due to broken symmetry in our sample commences at low-magnetic fields. Figure 5c illustrates the start of *σ*_xy_ transition to *ν* = 0 phase at CNP occurring at below 1 T. Collectively, these desirable characteristics (common among BGB devices made using our technique, see Supplementary Note 10 and Supplementary Fig. 10) are evidence of the graphene cleanliness in the BGB device structure.

## Discussion

Our study establishes an efficient strategy for the high-yield construction of diverse vdW heterostructures. A simple interface engineering scheme for modifying the exfoliation substrate with a sub-5 nm PVA is the key to this advance. This scheme enabled us to optimize the exfoliation step independently of the layer transfer step and vice versa. This ability resulted in two major outcomes. First is a consistent exfoliation method for producing large monolayer flakes. The second outcome is a high-yield layer transfer of exfoliated flakes without requiring a specialized transfer stamp. By using graphene as a model material, we further established the remarkable material and electronic properties of the resulting heterostructures.

We attribute the remarkable improvements of the PAGE technique over the conventional exfoliation method to a stronger adhesion of graphene to PVA than to SiO_2_. This hypothesis is confirmed by our observation that, when performing layer transfer at the glass transition temperature of PVA, a PVA-coated PDMS stamp can reliably peel off monolayer graphene that has been exfoliated directly on SiO_2_ (see Supplementary Note 8, and Supplementary Fig. 8).

Our extensive material and electrical characterizations confirmed the ability of our technique to produce heterostructures with clean interfaces. However, our layer assembly experiments revealed two crucial insights. First, we found that a lamination temperature of <110 °C is inadequate for achieving blister-free heterostructures prepared using PAGE. One possible explanation is that the exposure to PVA and water negatively impacts the surface properties of graphene. While we do not rule out this possibility, we point out that heterostructures prepared in ambient air using a polymer-free layer assembly could also suffer from a similar issue (e.g., see ref. ^18^ and Supplementary Fig. 21). These observations highlight the critical role of the lamination process on the interface quality of the resulting heterostructures.

The second insight is that subsequent annealing of the as-fabricated stacks in a UHV environment was highly effective in removing blisters. Indeed, our AFM and Raman measurements confirmed the cleanliness of the annealed heterostructures. However, one common observation about the high-temperature annealing is the accumulation of blisters^6^. Based on these two experimental insights, an important future direction for building heterostructures in ambient air is to explore the effectiveness of lamination at elevated temperatures in removing blisters—for example, using the process of Purdie et al. in ref. ^18^. The similarity between the glass transition temperatures of PMMA and PVA suggests that the removal of blisters by employing the lamination process of Purdie et al.^18^ should be feasible.

Past research has shown that performing the layer assembly in a moisture-free environment (e.g., in a glovebox) is another effective solution for producing heterostructures with clean interfaces^61,62^. However, the use of water for releasing exfoliated flakes in our current process limits its application for fabricating heterostructures in a glovebox environment. This pitfall could be resolved by replacing water with an anhydrous solvent for dissolving the PVA sacrificial layer (e.g., dimethyl sulfoxide^63^). Developing a glovebox-compatible process based on our technique constitutes an important future direction.

**Fig. 6:**
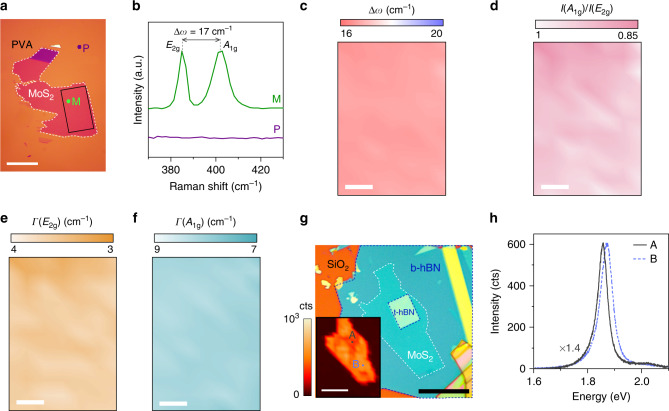
Fabrication of heterostructures from other layered materials. **a** Applying PVA- assisted exfoliation technique to MoS_2_ consistently yields large monolayer flakes. Scale bar is 20 μm. **b** Corresponding Raman spectra of “M” and “P” points in **a**. PVA does not have an overlapping Raman signature with MoS_2_, allowing the use of Raman spectroscopy for identifying the number of layers within the flake. **c**–**f** The spatial Raman maps of the flake in the **a** indicate the uniformity of its structural properties. The map was taken from the region inside the flake marked with the solid black box. In these plots, Δ*ω* is the spacing between *E*_2g_ and *A*_1g_ peaks, *I*(*A*_1g_)/*I*(*E*_2g_) is the ratio of the peak intensity, Γ(*E*_2g_) and Γ(*A*_1g_) are the FWHM of the peaks. Scale bars are 5 μm. **g** The optical image of a monolayer MoS_2_ encapsulated in hBN. The inset shows the spatial map of the PL peak intensity. Scale bars are 20 μm. **h** The representative PL spectra of the monolayer MoS_2_ at the encapsulated region (point “A”) and the exposed region (point “B”). The red-shift of the PL spectrum suggests the presence of a small tensile strain within the encapsulated region. The FWHM of the “A” and “B” spectra are ≈46 and 54 meV, indicating the high material quality of the exfoliated MoS_2_.

Our methodology can be generalized for constructing heterostructures from other layered materials besides graphene. We demonstrated the application of our exfoliation and layer transfer methods to MoS_2_, a material system that belongs to the large family of transition metal dichalcogenides. Applying our exfoliation technique resulted in large (<1000 μm^2^) monolayer MoS_2_ (Fig. 6a). This is an improvement over the conventional method, which is known to suffer from poor yield and small flakes (<300 μm^2^)^21^. We used Raman spectroscopy for identifying the number of layers within the candidate MoS_2_ flakes chosen through optical inspection. Figure 6b shows a typical Raman spectrum of the MoS_2_ flake in Fig. 6a (taken at point “M”) against the spectrum of the PVA background (taken at point “P”), indicating a monolayer flake. The Raman data indicate the broadening of the *A*_1g_ peak, the reduction of its intensity relative to the *E*_2g_ peak, and the smaller-than-expected spacing between these peaks (i.e., a theoretical separation of 20 cm^−1^ for unstrained monolayer MoS_2_^64^). These signatures of the Raman data suggest the presence of substrate-induced strain and doping in the as-exfoliated flake residing on PVA^64–68^. Decoupling the quantitative effects of doping and strain requires additional systematic studies. The spatial Raman map data (taken from the region marked with the solid black box in Fig. 6a) indicate the uniformity of the structural properties across the flake (see Fig. 6c–f).

We also demonstrated the utility of our layer transfer method for producing a stack of hBN/MoS_2_/hBN. In the resulting stack (Fig. 6g), the monolayer MoS_2_ is partially encapsulated in hBN; a large region of MoS_2_ remains exposed. This configuration gives additional degrees of freedom for building advanced device structures. For example, one can stack superconducting materials on the exposed regions of the semiconducting layered material to build Josephson junction transistors. Lastly, the strong intensity and the narrow FWHM of the photoluminescence (PL) spectrum of the stack in Fig. 6h indicates the high material quality of the monolayer MoS_2_.

Our construction method simplifies the fabrication of vdW heterostructures. Compared to the conventional method, our exfoliation method requires a significantly smaller number of attempts for producing the same number of exfoliated flakes. Further, our method enables the long-term storage of the exfoliated flakes for future use. The reason is that our layer transfer process relies on the removal of PVA for releasing the layered material. We fabricated a BGB device from a 3-week-old PAGE sample that showed a mobility on-par with fresh samples (see Supplementary Note 5). This contrasts with our experience with the layer transfer of exfoliated flakes on SiO_2_, where we could not mechanically detach monolayer flakes from SiO_2_ after a few days of storage. Lastly, given their consistent results and versatility to different layered materials, our exfoliation and the layer transfer methods can be standardized, hence opening the door for building automated machines to perform those steps. The collective effects of the above features of our construction method enable a high-yield production of vdW heterostructures.

## Methods

### Preparation of PVA-coated substrates

In all experiments in this study, we used a dilute PVA solution with a concentration of 3% wt vol^−1^. We prepared the solution by dissolving the PVA powder (MW = 9000, Sigma) in deionized water. Prior to the exfoliation experiments, SiO_2_/Si substrates (285 nm oxide) were cleaned with a Piranha solution, followed by the spin coating of the PVA solution at a spin speed of 8000 rpm for 30 s. The PVA film was not baked after the spin coating and prior to the exfoliation step. We measured the thickness of the PVA film by fitting the ellipsometry data using the Cauchy model. The ellipsometry (J. A. Woollam) was done in the visible wavelength range and at three different angles. The Cauchy model consistently provided a refractive index of 1.46 ± 0.02, which is in good agreement with the known refractive index of PVA. The typical measured PVA film thickness using this technique is about 3.2 nm.

### Graphene exfoliation

We prepared the bulk crystals of graphite on a scotch tape (3 M, Cat. 105). After placing the tape on the PVA-coated substrate at room temperature, we performed a brief heat treatment at 85 °C for 10 s on a hotplate. We observed no apparent improvements in size or yield of the exfoliated flakes with longer heat treatments. Before detaching the tape, the sample was removed from the hotplate. The tape was then peeled back slowly (at a speed of 2–3 mm s^−1^), starting from one end of the substrate. While we had no tight control over the exfoliation angle, we targeted for an angle below 30°.

### Preparation of the polymeric stamp and layer transfer

We followed the procedure in ref. ^16^ to produce the PDMS stamps. These stamps were then modified by applying a PPC coating, prepared following the procedure in ref. ^15^. To locally dissolve PVA during the layer transfer, a water drop was injected using a manual syringe assembly, as shown in Supplementary Note 2, and Supplementary Fig. 2. The substrate temperature was 40 °C during this step. After transferring the flake onto the stamp, the stamp was immersed in a deionized water bath at 40 °C for about 1 h, while changing the DI water every 20 minutes. The stamp was then dried using gentle N_2_ flow, then left in a desiccator for later use.

### Ultra-high-vacuum (UHV) annealing step

The UHV annealing for all stacks was done at 400 °C for 2 h. The base pressure of the system was about 1 × 10^−10 ^mbar. The ramp-up rate of the temperature was 5 °C min^−1^ up to 150 °C and was 10 °C min^−1^ after that.

### Fabrication of gated-Hall bar devices

Two-dimensional metal contacts were formed on graphene for both GB and BGB structures using a combination of electron-beam lithography (EBL), e-beam metal evaporation (Cr 5 nm/Au 50 nm), and metal lift-off. The device structure was complete after defining the active region using a combination of EBL and reactive ion etching.

### Raman spectroscopy and strain calculation

Raman measurements were made using the Horiba Xplora micro-Raman system with a 532 nm laser. The G and 2D lines were fitted with a single Lorentzian function. The uniaxial strain-sensitivity of the G mode (i.e., Δ*ω*/Δ*ε* = 23.5 cm^−1^/%) was used for calculating the residual strain^39^.

### Electronic transport measurements

We used low-current, low-frequency lock-in techniques for measuring the longitudinal resistance (*R*xx) and transverse Hall resistance (*R*xy) of the graphene Hall bar devices. Low-temperature measurements were made in a cryogen-free micro-manipulated Lakeshore probe station, CRX-VF. Low-temperature measurements below 9 K were made in a cyro-free superconducting magnet system, TeslatronPT (see Supplementary Fig. 10).

## Supplementary information

Supplementary Information

Description of Additional Supplementary Files

Supplementary Movie 1

## Data Availability

The authors declare that the data supporting the findings of this manuscript are available within the article and its Supplementary Information files. Extra data are available from the corresponding author upon reasonable request.
