# Supplementary material for: Versatile construction of van der Waals heterostructures using a dual-function polymeric film
Source: Nat Commun. 2020 Jun 15;11:3029. doi: 10.1038/s41467-020-16817-1 (PMC7295972; doi:10.1038/s41467-020-16817-1)
Supplement: Supplementary file 1 — Supplementary Information [file 41467_2020_16817_MOESM1_ESM.pdf]

Supplementary Information

Versatile construction of van der Waals heterostructures using a dual-function polymeric film

Huang and Alharbi *et al.*

Supplementary Note 1. PVA-assisted exfoliation of very large monolayer flakes. Our approach consistently produces very large monolayer graphene flakes and is applicable to different layered materials. In Supplementary Fig. 1, we show example optical images for monolayer flakes of graphene and MoS_2, produced using our PVA-assisted exfoliation method.

Supplementary Note 2. Layer assembly setup. In Supplementary Fig. 2, we show a photo of our custom-made layer assembly setup, consisting of a transfer stage and a water injection setup. The hemispherical polymeric stamp prepared on the glass slide is held by a micro-manipulator over the exfoliation substrate. To release the exfoliated flakes, we manually inject a small drop of water using a syringe with a 27 G needle close to the landing region. In the Supplementary Movie 1, we show an example release process, in which a PPC stamp was used to pick up monolayer graphene.

Supplementary Note 3. Room-temperature resistivity as a function of carrier density. In Supplementary Fig. 3, we show the zoomed-in plot of the sheet resistivity (ρ_{xx}) for the BGB-1 device at room temperature as a function of applied gate bias. The data show that the device has a room-temperature sheet resistivity below 40 Ω at large carrier densities $n \sim 2 \times 10^{12}$ cm^{-2}.

Supplementary Note 4. Calculation of theoretical acoustic-phonon-limited mobility. The acoustic phonon limit at room temperature is calculated using (Ref. [1])

$$\mu = \frac{3.7 \times 10^7}{D^2 \cdot \tilde{n}} \qquad (1)$$

where D is deformation potential in units of eV and $\tilde{n}$ is the carrier density in units of 10^{12} cm^{-2}. We used D = 14 eV in our calculation, which corresponds to a theoretical electron-phonon resistivity $\rho_{e\text{-}ph} = 32\ \Omega$ (Ref. [2]).

Supplementary Note 5. Long-term storage of PAGE samples. Our technique allows the release of flakes after long-term storage. To demonstrate this capability, we fabricated a graphene encapsulated in hBN (BGB) device. In Supplementary Fig. 4, we show the optical image and the electronic measurements of this BGB device (BGB-3 in supplementary Fig.4). In this experiment, we first exfoliated graphene using PAGE. The PAGE sample was then stored inside a desiccator for 3 weeks. We then used a monolayer graphene flake on this PAGE sample for building the BGB device.

The room-temperature mobility is ~125,000 $cm^2\ V^{-1}\ s^{-1}$ at low carrier densities. We note that this device was damaged during a room-temperature measurement and prior to the low-temperature measurements. Notice the broadening of the ρ_{xx} at the n-branch and at around the CNP in the low-temperature ρ_{xx}. The green curve in Supplementary Fig. 4 shows the room-temperature ρ_{xx} of this device. Despite this issue, we were able to measure the hole branch mobility of 800,000 $cm^2\ V^{-1}\ s^{-1}$ at 9 K. Due to the broadening of the ρ_{xx} at the electron branch and near the charge neutrality point (CNP), the estimation of the carrier inhomogeneity is unreliable for this device.

Supplementary Note 6. Summary of the low-temperature transport measurements on graphene heterostructures. To demonstrate the high quality of graphene produced by our method, we fabricated several devices from graphene-on-hBN (GB) and graphene encapsulated in

hBN (BGB) stacks. We evaluated the electronic characteristics of graphene using four-point measurements for multiple devices and applying low-current, low-frequency lock-in amplifier techniques. In Supplementary Fig. 5 and Supplementary Fig. 6, we show the measurement results of the longitudinal resistivity (ρ_{xx}), graphene conductivity ($\sigma = 1/\rho_{xx}$), low-temperature mobility (μ), and the carrier inhomogeneity (n^*) for GB and BGB devices, respectively. In Supplementary Table 1, we also provide a summary of the electronic properties of the BGB devices. We also provide the details of the device structure, namely the thickness of the bottom hBN dielectric and global versus local gating. These measurements were made at 9-10 K (the base temperature of our measurement system).

Supplementary Note 7. Effects of thermal cycles on room-temperature mobility. BGB-1 device shows very high mobility at room temperature compared to the other BGB devices in our study. This difference can be explained by the fact that the BGB-1 device is the only one that underwent a thermal cycle in the measurement system. Thermal cycling has been previously observed to improve the mobility of BGB devices (see Ref. [4]). The temperature sequence of BGB-1 (shown in Supplementary Table 2) was planned to protect the device to ensure that we obtain a full set of measurement data, but we did not expect it to improve the room-temperature mobility.

Supplementary Table 2. shows the history of electrical measurements made on BGB-1. All measurements were made in a Lakeshore probe station without breaking the vacuum. The first mobility data (before the thermal cycle) at 300 K is comparable to the state-of-the-art devices (Ref. [3]). However, the mobility at 300 K in subsequent measurements (after the first thermal cycle) is

noticeably higher. We noticed this improvement during the third measurement (i.e. after the first cooling). Hence, we performed an additional thermal cycle to see whether mobility changes further. As can be seen in Supplementary Fig. 7, the mobility remained nearly unchanged after performing an additional thermal cycle.

Previous studies have reported the improvement of the carrier mobility in BGB devices after thermal cycling in the measurement system and attributed this phenomenon to the modulation of the charge inhomogeneity during the thermal cycles (Ref. [4]).

Supplementary Note 8. Layer assembly using PVA-coated stamps. We hypothesized that graphene has stronger adhesion to PVA than to SiO_2. To test this hypothesis, we coated the PDMS stamp with PVA and examined whether this stamp can detach graphene flakes exfoliated directly on SiO_2 substrates. Supplementary Fig. 8(a) shows the schematic illustration of the experiment. Indeed, we found that the PVA stamp can detach graphene from SiO_2 when the layer transfer process is done at about the glass transition temperature. This observation confirms our hypothesis.

We then used the transferred graphene flakes using the PVA-coated stamp for building GB structures. Note that in these structures, both monolayer graphene and hBN flakes were produced using the conventional exfoliation method. Supplementary Fig. 8(b) shows the optical image of a back-gated four-point device. Supplementary Fig. 8(c)-(d) show the electrical characterization results for a GB device. This device showed an estimated charge inhomogeneity of around 4×10^{10} cm^{-2} and a low-temperature carrier mobility of 40,000 $cm^2\ V^{-1}\ s^{-1}$ at low densities. The

use of a PVA-coated stamp for layer transfer process also adds to the library of methods for building vdW heterostructure from layered materials exfoliated directly on SiO_2.

Supplementary Note 9, Extraction of hBN dielectric constant. Precise estimation of the hBN oxide capacitance (C_{ox}) is critical to the proper extraction of μ and n^*. For this, one must determine the dielectric constant of hBN and its thickness. The evaluation of C_{ox} is then straightforward using $C_{ox} = \varepsilon_r\varepsilon_0/t_{hBN}$, where ε_r, ε_0, and t_{hBN} are the dielectric constant of hBN, permittivity of free space, and the hBN thickness.

We extracted the dielectric constant of hBN by plotting the carrier density as a function of the back-gate bias (Supplementary Fig. 9). In particular, we used the magneto-transport measurements of the BGB-4 device to directly determine the carrier density at a few different back-gate biases (V_g) using $n = (eB_v/h)\,\nu$, where B_v is the magnetic field at which the filling factor index ν takes place, e is the elementary charge, and h is the Planck's constant. Notice that for this analysis we extracted the charge densities at sufficiently high carrier densities where the effect of quantum capacitance is negligible. From the linear fit to the data we found $\varepsilon_r = 3$.

Supplementary Note 10. Magneto-transport measurement of BGB-4 device at 1.5 K. In Supplementary Fig. 10, we show the magneto-transport measurement of BGB-4 device at 1.5 K in a magnetic field up to 12 T. The well-resolved quantum Hall states (QHS) indicate the good quality of the graphene. The onset of Shubnikov de Haas (SdH) coincides with a field of ~250 mT. The lifting of zero energy level degeneracy starts below 2 T.

Supplementary Note 11. AFM phase imaging on PVA-exposed graphene. To study the effect of PVA exposure, we performed high-resolution AFM measurements on graphene exfoliated on SiO_2 substrates. The main reason behind this experimental design is to examine how exposure to water and PVA impacts the surface properties of graphene. However, when graphene resides on a stamp, there are multiple non-ideal interfaces within the stack, which make reliable interpretation of the AFM difficult. To better illustrate this, in Supplementary Fig. 11, we show the AFM images of graphene on a t-hBN/PPC stamp (i.e., t-hBN is sandwiched between graphene and PPC). These images were obtained in a non-tapping mode, because of difficulties in performing tapping mode AFM on a soft stamp. Notice that the graphene region on t-hBN shows more features in these AFM images than the graphene region outside the t-hBN. It is, however, difficult to determine the origin of these features, whether they originate from the surface of graphene, or the graphene-hBN interface, or the hBN-PPC interface. Moreover, we cannot rule out that the line-like features in these images are due to the real-time damage to the graphene film by the AFM tip since the graphene film is not supported firmly by the PPC. Doing the AFM imaging experiments on freshly exfoliated graphene flakes on SiO_2 allows us to directly attribute the observations to the effect of PVA and water on the surface properties of graphene. Further, the firm support by SiO_2 substrate allows us to perform AFM imaging in tapping mode.

In all experiments, we prepared exfoliated graphene samples one day before AFM measurements. Below, we describe the details of the experiments and major observations. Supplementary Table 3 gives a summary of the measurements.

(a) Presence of moisture on the surface of as-exfoliated graphene. In Supplementary Fig. 12, we show the AFM topography and phase images of an as-exfoliated graphene flake before and after the hotplate bake. We observed noticeable variations in the phase image of the flake before the hotplate bake. We note that this observation was consistent among different graphene flakes. We suspected that the observed variations in the phase image arise from the presence of moisture on the graphene. Indeed, those features in the AFM phase image vanished after a brief bake of the samples on a hotplate at ~110 ˚C for 5 minutes, as shown in Supplementary Fig. 12(d). This observation indicates a potential challenge for obtaining blister-free stacks when performing the layer assembly in ambient air. In particular, the trapping of moisture could result in the formation of blisters at the interface between the stacked materials.

(b) Phase difference between PVA film and graphene. Supplementary Fig. 13 shows the AFM topography and phase image on a PAGE sample. Phase imaging is useful for distinguishing materials based on their hardness (i.e., Young's modulus). Given the noticeably larger Young's modulus of graphene than PVA, the phase imaging is suitable for studying the effect of graphene exposure to PVA. However, the phase value depends on the set-point amplitude ratio (r_{sp}) during the AFM measurements (Ref. [5]). Note that in our AFM studies below, we plotted the phase of the tapping mode cantilever. In our experiment, graphene shows a larger phase value in the AFM image than the PVA when $r_{sp} < 0.3$. However, when $r_{sp} > 0.3$, we observe the opposite of this trend to occur.

In panel (a), the PVA film region shows higher rms roughness compared with monolayer graphene. In panel (b), we plot the phase of the tapping mode cantilever. As can be seen, it is possible to

distinguish the PVA from graphene based on their mean phase value (φ), which is lower for PVA than the monolayer graphene since r_{sp} = 0.1.

(c) Increase in the phase of graphene after exposure to PVA and water. Supplementary Fig. 14 - 18 include AFM topography and phase images from 5 different flakes before and after the exposure to PVA. In those experiments, graphene flakes were first exfoliated on the pre-cleaned SiO_2/Si substrate (i.e. conventional exfoliation). The samples were baked at 110 ˚C for 5 minutes prior to AFM measurements. The purpose of this step is to remove the moisture from ambient air and obtain images for as-exfoliated graphene flakes. Then, samples were spin-coated with a thin PVA film and baked around the glass transition temperature of PVA on a hotplate for 10 seconds to mimic the brief heat treatment during our exfoliation process. Following a thorough water rinse, samples were first dried by gentle N_2 flow and baked at 110 ˚C for 5 minutes. Finally, AFM measurements were performed to obtain images after exposure to PVA and water rinse. The samples after exposure to PVA and water show higher average roughness and phase value (Phase imaging was performed with r_{sp} > 0.3). The results suggest the negative impact of exposure to PVA and water on the surface properties of graphene.

To determine whether the changes in the AFM images of graphene are exclusively due to the exposure to PVA, we performed a controlled experiment. In this experiment, we did not apply the PVA coating on graphene. Specifically, after the first AFM imaging, we immersed the graphene sample in water, followed by a gentle N_2 blow-dry and the hotplate bake at 110 ˚C. Interestingly, the changes in the AFM phase and topography images of graphene in this experiment were

comparable to those involving graphene exposure to the PVA coating. (see Supplementary Fig, 19 – 20).

Supplementary Note 12. Controlled experiment of the lamination process. The BGB stacks made using our PVA-based technique generally contain blisters after the lamination step. To understand the origin of this issue, we performed a controlled experiment. In this experiment, we built a BGB stack in which we used a monolayer graphene flake directly exfoliated on SiO_2. Except for this modification, all other processing details were identical to the BGB fabrication using PAGE samples. Note that unlike monolayer graphene obtained using PAGE, the monolayer graphene in this experiment was not exposed to polymer or solvent during the stack fabrication. Supplementary Fig. 21 shows the side-by-side optical images of BGB stacks obtained using our method (Supplementary Fig. 21(a)) and using the polymer-free layer assembly (Supplementary Fig. 21(b)). We found that the resulting BGB stack from this controlled experiment also suffers from excessive blisters. This observation is consistent with previous studies (e.g., see Ref. [3]) that also reported the formation of blisters in polymer-free layer assembly of graphene stacks. This experiment indicates that the lamination process used in our stack fabrication process is sub-optimal.

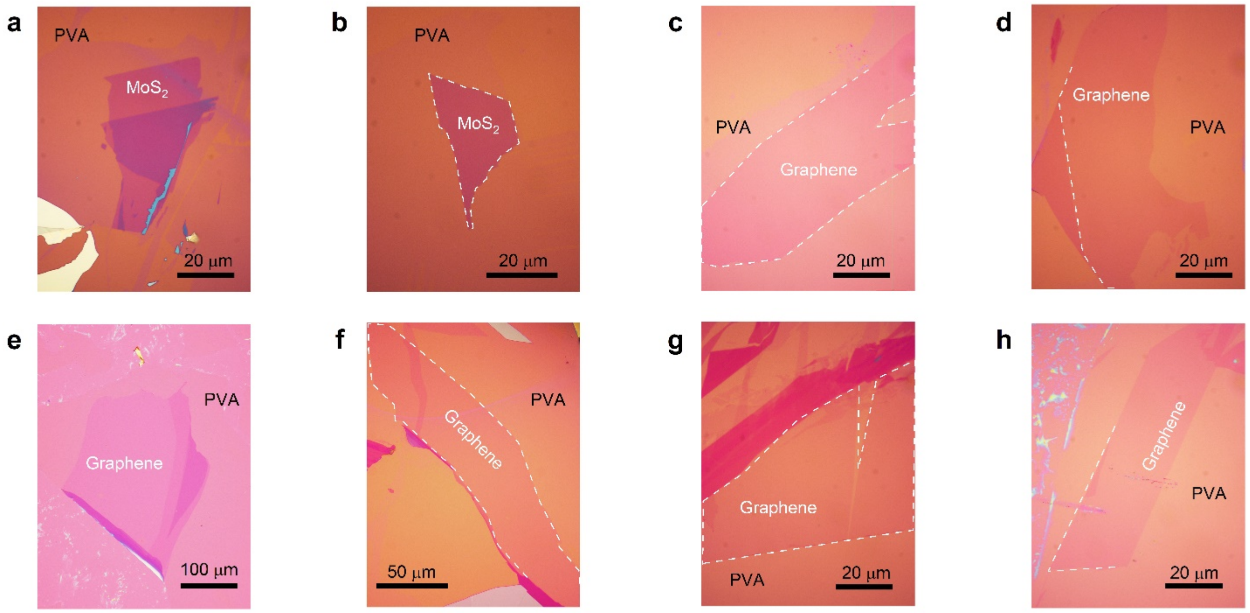

Supplementary Fig. 1: Representative optical images of a few additional (a),(b) monolayer MoS_2 and (c)-(h) monolayer graphene flakes produced using the PVA-assisted exfoliation method. Our method consistently produces very large flakes.

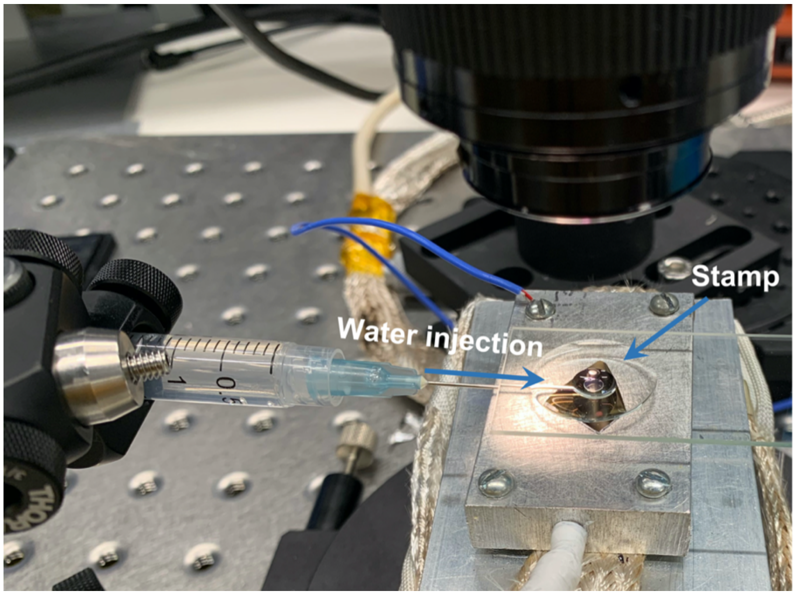

Supplementary Fig. 2: Optical image of our custom-made layer transfer and water injection setup. The syringe is assembled on a stand with X and Y micromanipulators, providing precise control over the position of the needle. Water is injected manually using the syringe.

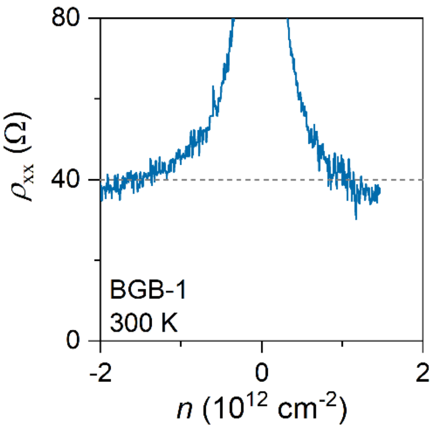

Supplementary Fig. 3: Room-temperature longitudinal resistivity of BGB-1 device indicates the remarkable properties of graphene.

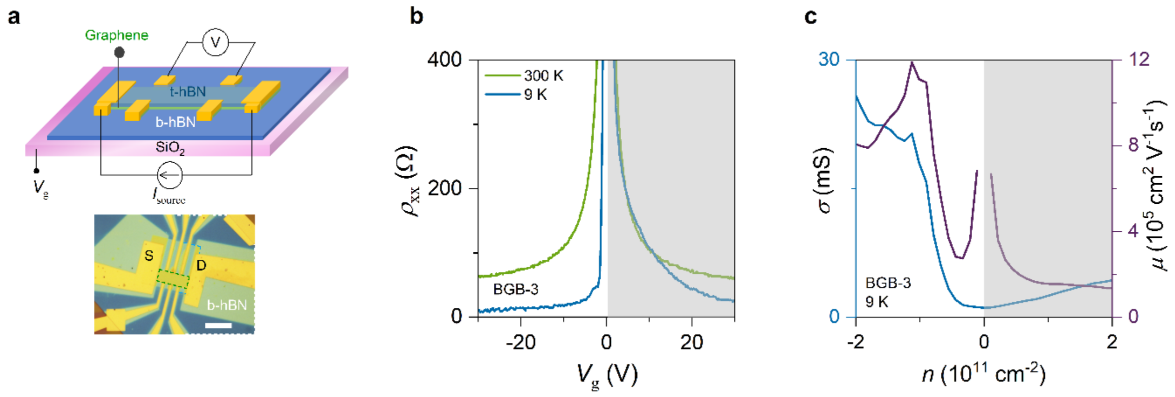

Supplementary Fig. 4: (a) Schematic illustration and optical image of the BGB-3 device. The PAGE sample was stored for three weeks before it was used for device fabrication. Scale bar is 10 μm. (b) Longitudinal resistivity measured at room temperature and 9 K. The low-temperature data show the damage to the n-branch. (c) The device gives peak hole mobility of ~800,000 cm^2 V^{-1} s^{-1} at 9 K.

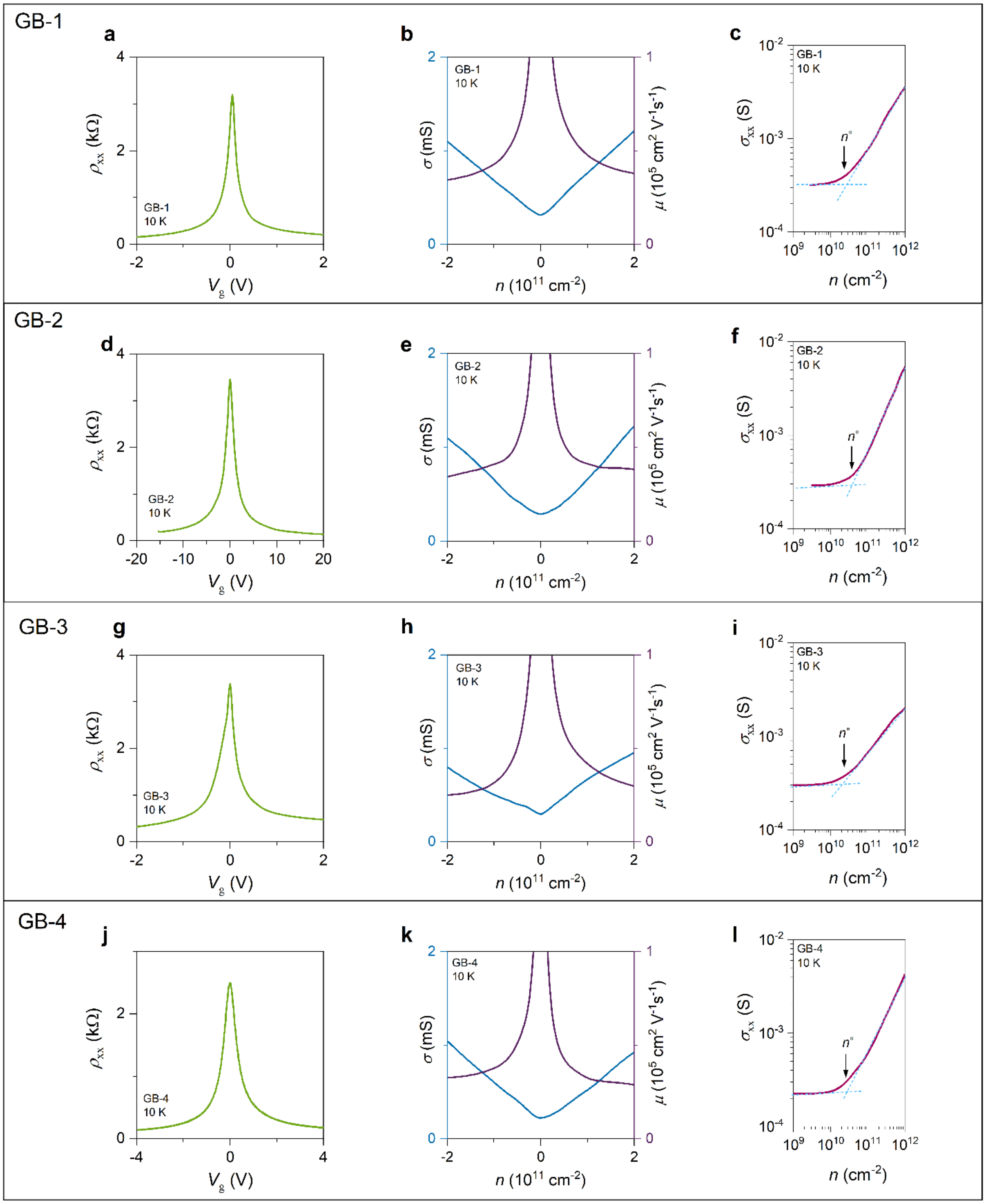

Supplementary Fig. 5: Electronic transport measurements of multiple GB devices: (a)-(c) GB-1, (d)-(f) GB-2 device, (g)-(i) GB-3 device, and (j)-(l) GB-4 device. For building these devices, we produced the monolayer graphene using PAGE.

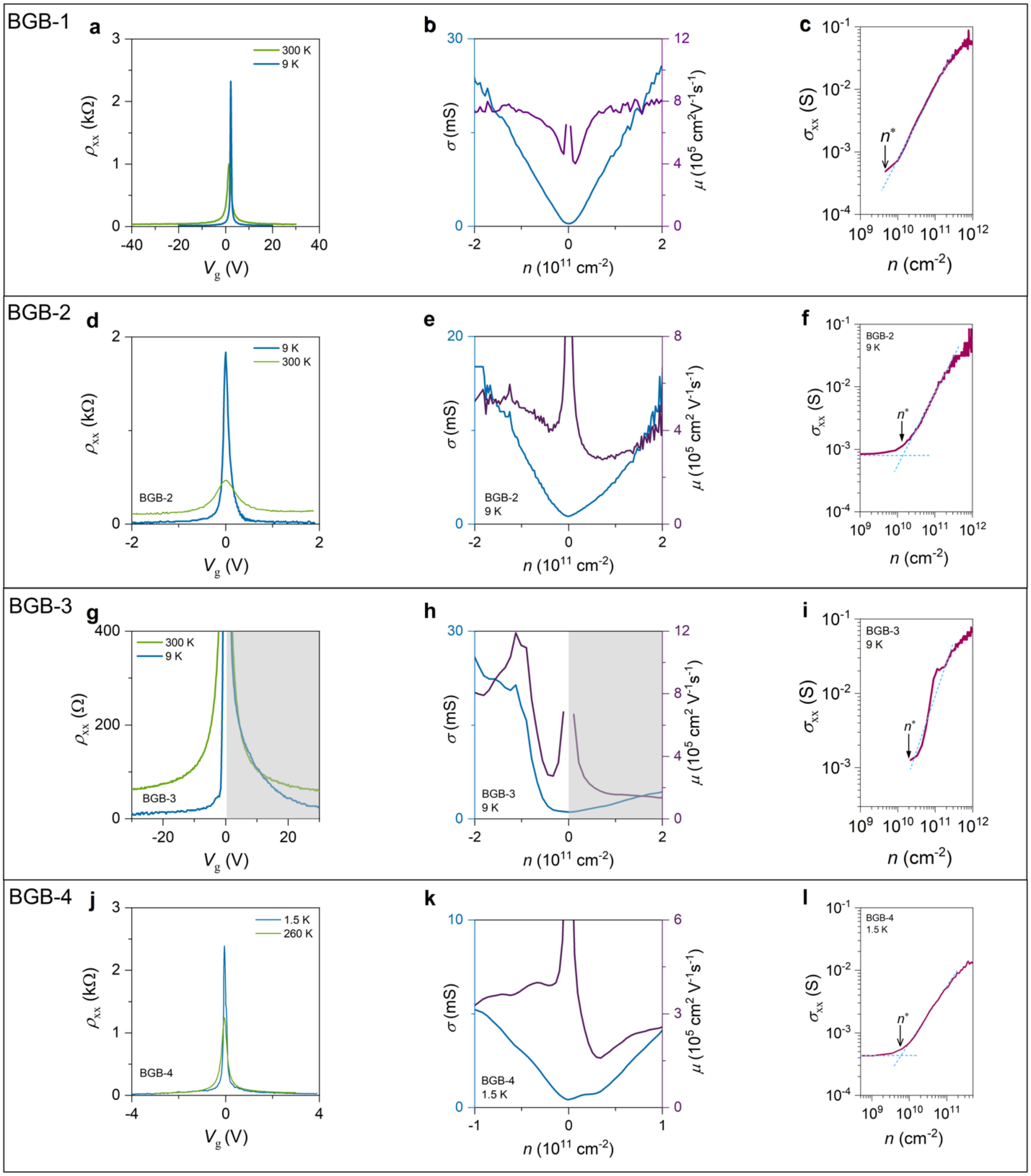

Supplementary Fig. 6: Electronic transport measurements of multiple BGB devices: (a)-(c) BGB-1, (d)-(f) BGB-2 device, (g)-(i) BGB-3 device, and (j)-(l) BGB-4 device. For building these devices, we produced the monolayer graphene using PAGE. Note that the estimation of carrier inhomogeneity of BGB-3 is unreliable because of the CNP broadening due to the electron branch damage.

Device ID	W (μm)	L' (μm)	t_{bBN} (nm)	μ (cm^2 V^{-1} s^{-1}) 300 K	μ (cm^2 V^{-1} s^{-1}) 9 K	n^* (cm^{-2})	Gate
BGB-1	6	6	91	146,000<br>250,000*	800,000	$< 5 \times 10^9$	Global back gate
BGB-2	4	2.8	25	175,000	500,000	$< 9 \times 10^9$	Local back gate
BGB-3	6	6	67	125,000	800,000	--	Global back gate
BGB-4	4	1.8	26	140,000	360,000 (1.5 K)	$< 6 \times 10^9$	Local back gate

Supplementary Table 1. Summary of transport characteristics of BGB devices. We note the effect of thermal cycles in the measurement system on the room-temperature mobility of BGB-1 devices (see Supplementary Note 7). Other devices in our experiments did not undergo a thermal cycle.

Measurement No.	Temperature (K)	Gate voltage sweep range (V)	Mobility (cm^2 V^{-1} s^{-1})	Carrier density for reading mobility (cm^{-2})
1	300	-30 to 30	146,000	1.5×10^{11}
2	9	-10 to 10	795,000	1.6×10^{11}
3	300	-60 to 30	260,000	1.5×10^{11}
4	9	-20 to 20	810,000	1.4×10^{11}
5	300	-30 to 30	273,000	1.5×10^{11}

Supplementary Table 2. History of electrical measurements made on the BGB-1 device.

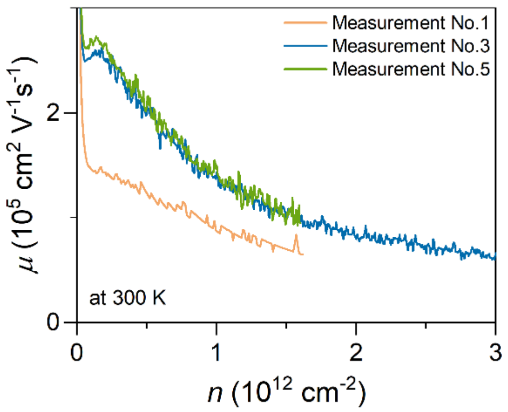

Supplementary Fig. 7: Comparison of the carrier-density dependent mobility of BGB-1 at room-temperature for different measurements. The measurement No. in this plot represents the conditions listed in Supplementary Table 2.

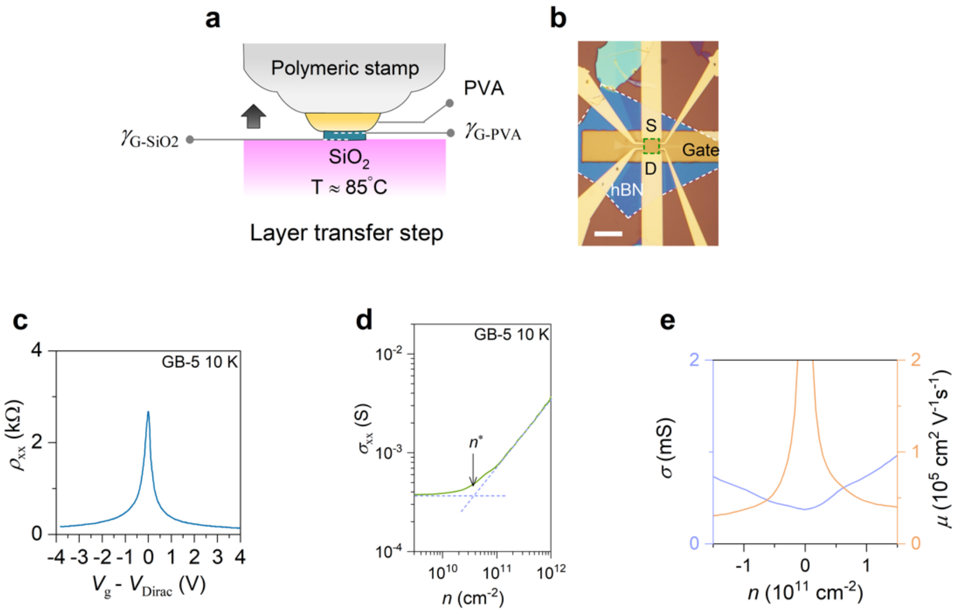

Supplementary Fig. 8: (a) Schematic illustration of the experiment in which the PDMS stamp was coated with PVA. (b) The optical image of a GB device made using this layer transfer technique. (c-e) The electrical characterization results of a GB device at 10 K.

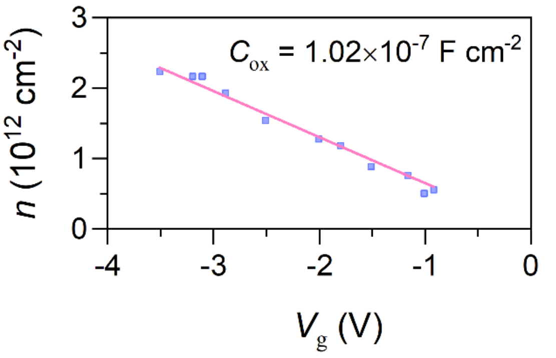

Supplementary Fig. 9: The plot of measured n as a function of the back-gate bias (V_g) obtained for the BGB-4 device. The carrier density was extracted directly from the magneto-transport characteristics of the device. The fit to the data gives a dielectric constant of 3.

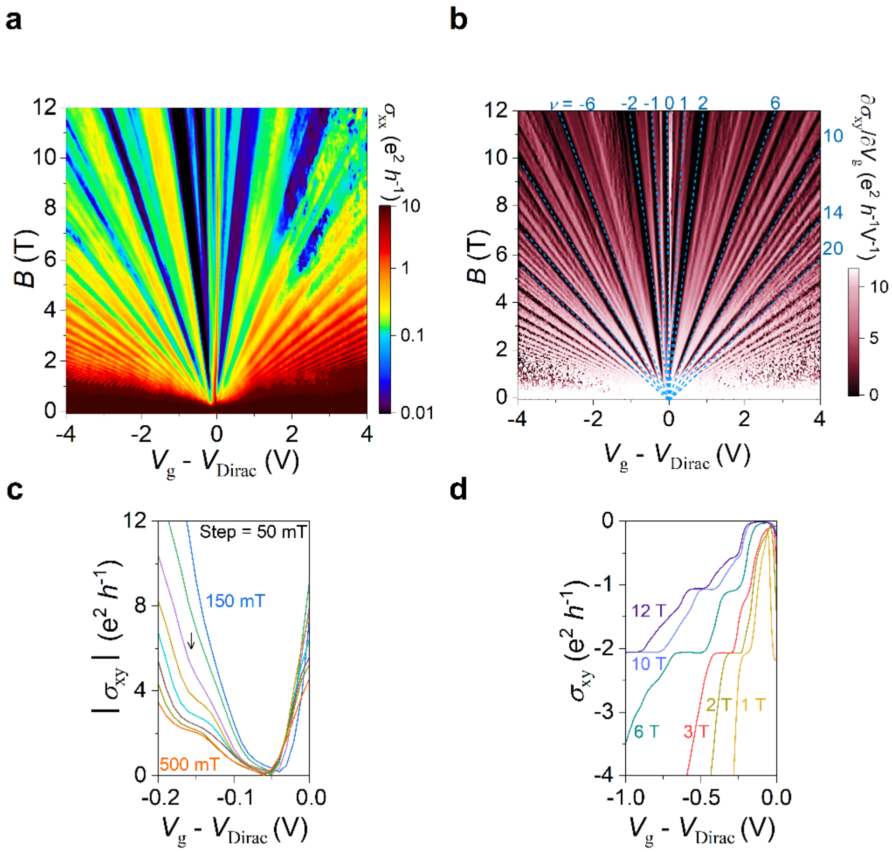

Supplementary Fig. 10: Magneto-transport measurement of BGB-4 at 1.5 K. (a) Fan diagram of longitudinal conductivity. (b) Hall conductivity with dashed lines showing the QHS. (c) The SdH starts at a low magnetic field about 250 mT. (d) The lifting of the zero level starts below 2 T.

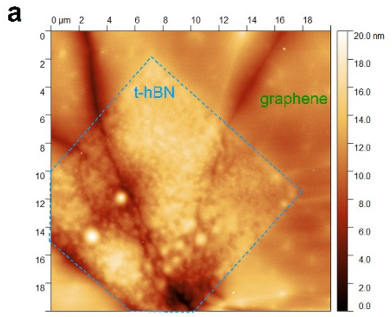

Supplementary Fig. 11: (a), (b) AFM topography and topography error images of graphene-BN-PPC. BN is highlighted by blue dashed lines. The size of the graphene flake is larger than the measurement window size.

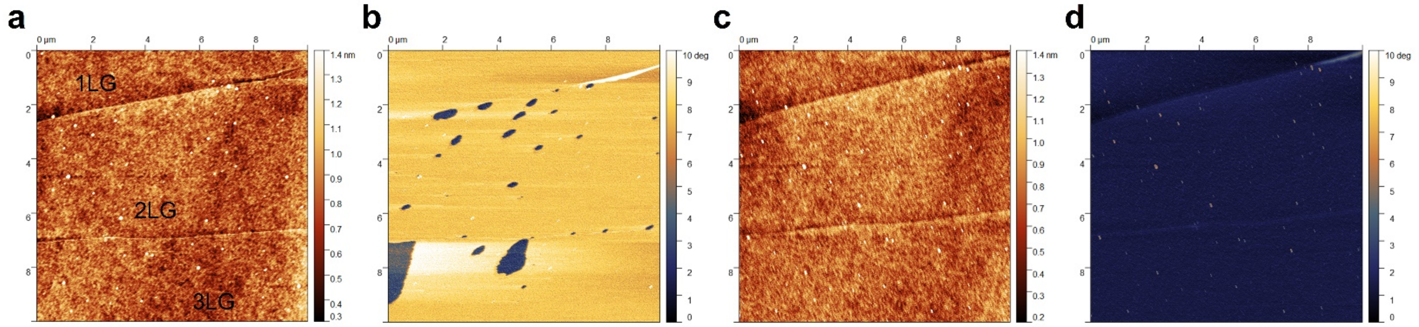

Supplementary Fig. 12: (a), (b) AFM topography and phase image of an example as-exfoliated graphene flake before hotplate bake (r_{sp} = 1). (c), (d) AFM topography and phase image of the same region of the flake after hotplate bake (S2 in Supplementary Table 3). The phase image after the hotplate bake is uniform and shows a smaller phase value than before the bake.

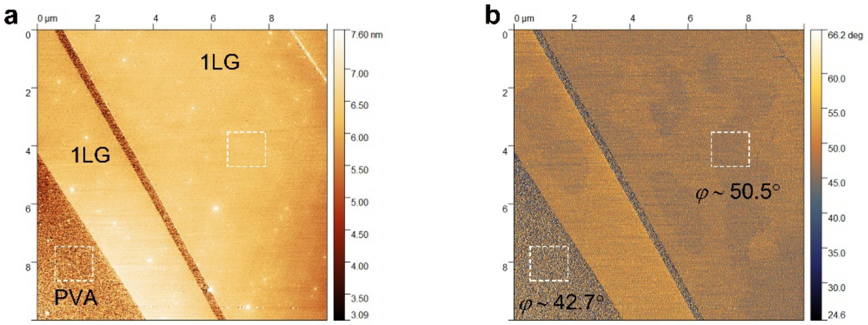

Supplementary Fig. 13: (a) AFM topography and (b) phase image of a PAGE sample (S1). R_q (root-mean-square (rms) of height variations) and φ (mean phase value) are calculated using data within the highlighted square regions. A summary of data for all flakes are given in Supplementary Table 3.

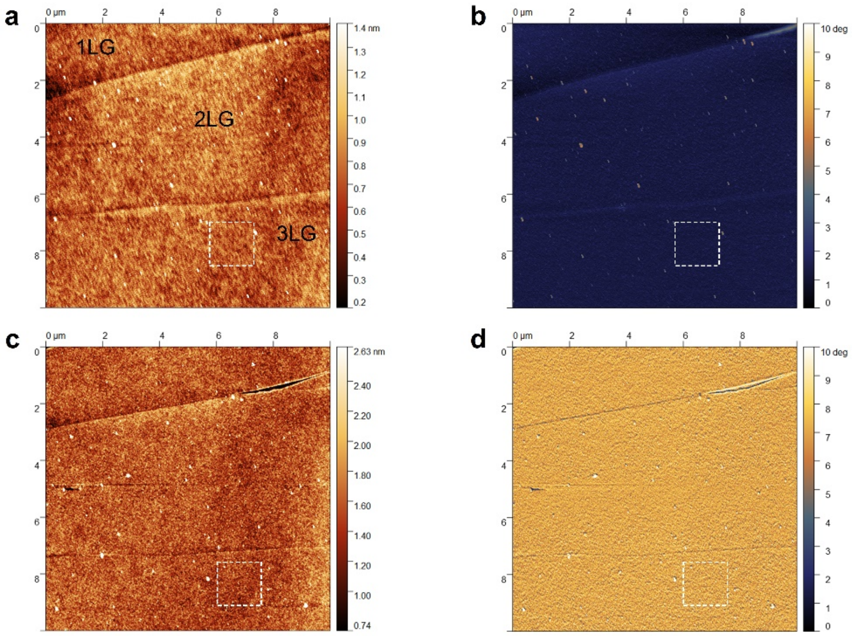

Supplementary Fig. 14: (a) AFM topography, (b) AFM phase image of an as-exfoliated graphene flake (S2). (c), (d) AFM images of the same region of the flake after exposure to PVA and subsequent water rinse. R_q (root-mean-square (rms) of height variations) and φ (mean phase value) are calculated using data within the highlighted regions, given in Supplementary Table 3.

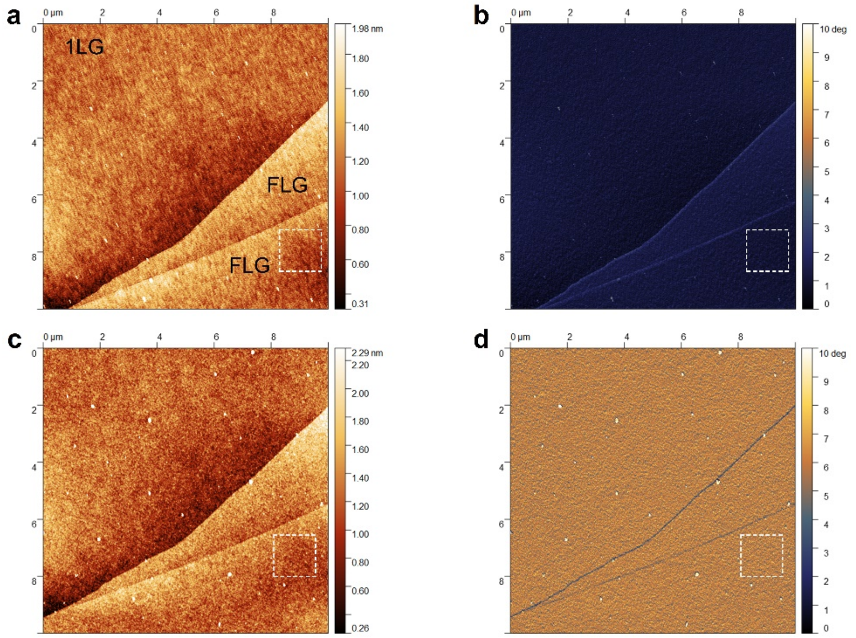

Supplementary Fig. 15: (a) AFM topography, (b) AFM phase image of an as-exfoliated graphene flake (S3). (c), (d) AFM images of the same region of the flake after exposure to PVA and subsequent water rinse. R_q (root-mean-square (rms) of height variations) and φ (mean phase value) are calculated using data within the highlighted regions, given in Supplementary Table 3.

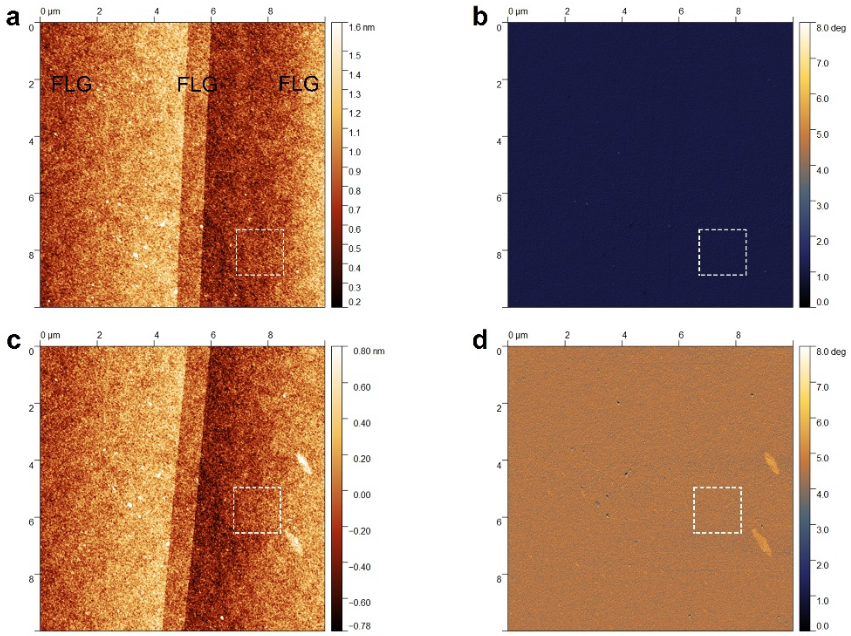

Supplementary Fig. 16: (a) AFM topography, (b) AFM phase image of an as-exfoliated graphene flake (S4). (c), (d) AFM images of the same region of the flake after exposure to PVA and subsequent water rinse. R_q (root-mean-square (rms) of height variations) and φ (mean phase value) are calculated using data within the highlighted regions, given in Supplementary Table 3.

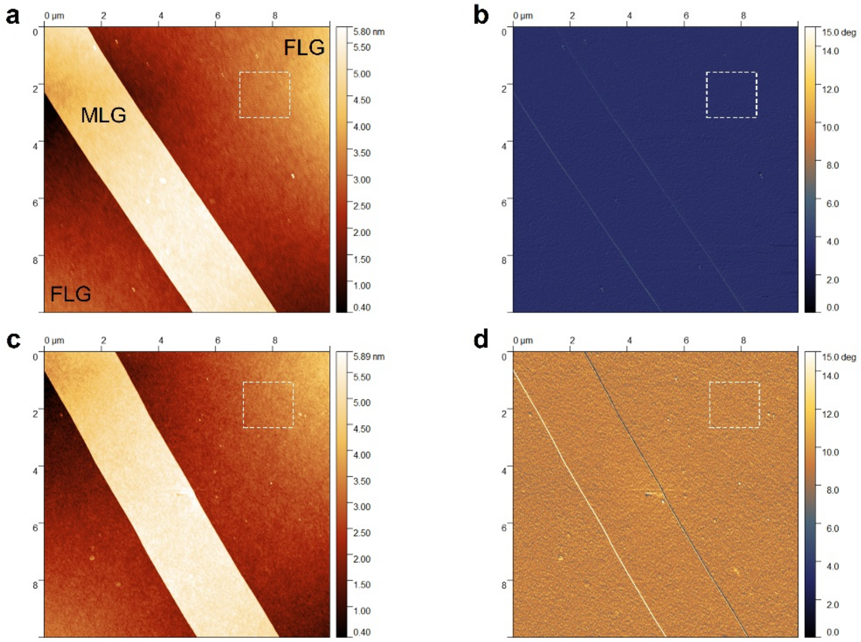

Supplementary Fig. 17: (a) AFM topography, (b) AFM phase image of an as-exfoliated graphene flake (S5). (c), (d) AFM images of the same region of the flake after exposure to PVA and subsequent water rinse. R_q (root-mean-square (rms) of height variations) and φ (mean phase value) are calculated using data within the highlighted regions, given in Supplementary Table 3.

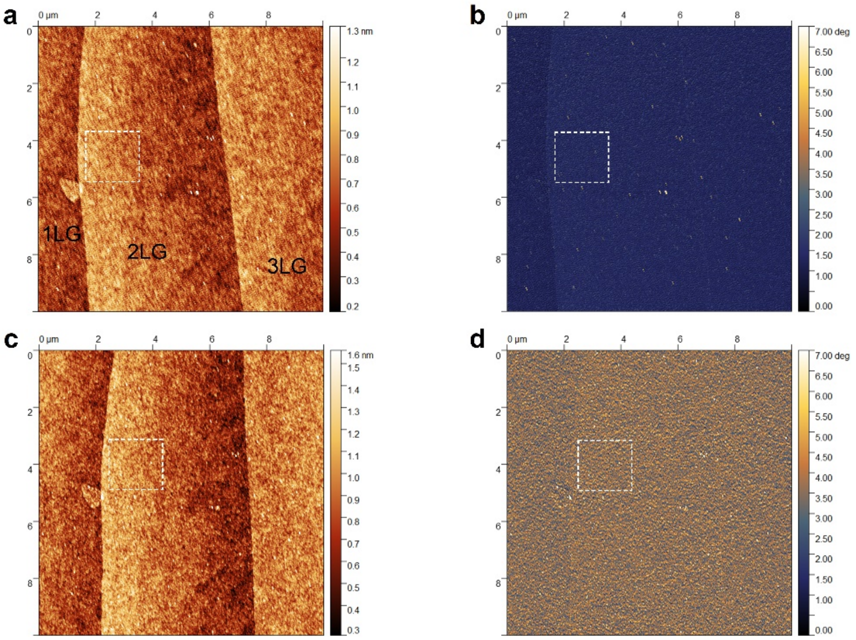

Supplementary Fig. 18: (a) AFM topography, (b) AFM phase image of an as-exfoliated graphene flake (S6). (c), (d) AFM images of the same region of the flake after exposure to PVA and subsequent water rinse. R_q (root-mean-square (rms) of height variations) and φ (mean phase value) are calculated using data within the highlighted regions, given in Supplementary Table 3.

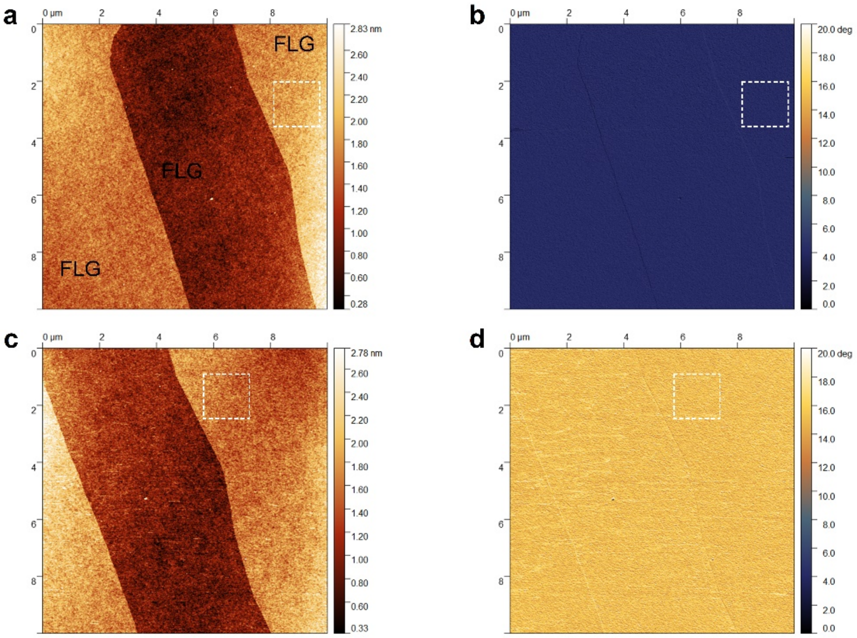

Supplementary Fig. 19: (a) AFM topography, (b) AFM phase image of an as-exfoliated graphene flake (S7). (c), (d) AFM images of the same region of the flake after exposure to water rinse. R_q (root-mean-square (rms) of height variations) and φ (mean phase value) are calculated using data within the highlighted regions, given in Supplementary Table 3.

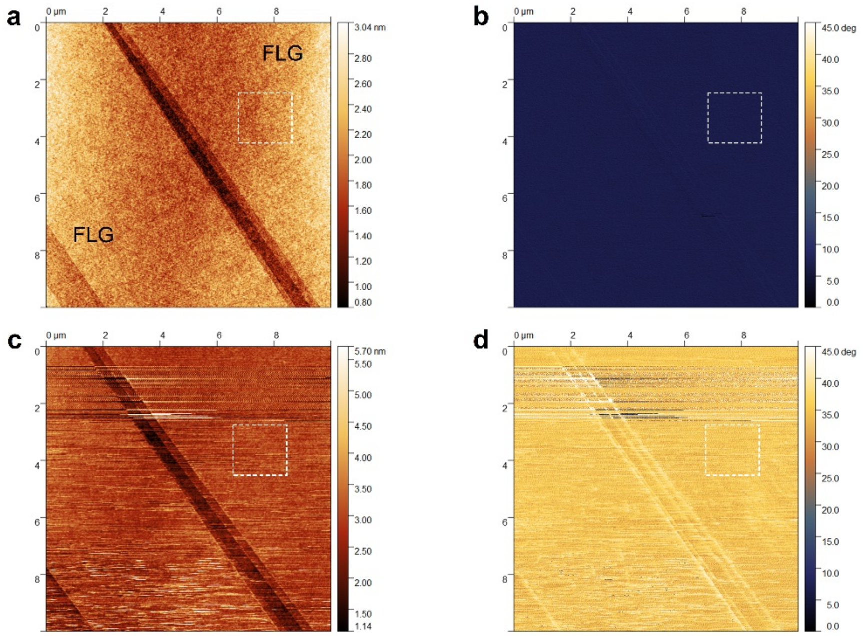

Supplementary Fig. 20: (a) AFM topography, (b) AFM phase image of an as-exfoliated graphene flake (S8). (c), (d) AFM images of the same region of the flake after exposure to water rinse. R_q (root-mean-square (rms) of height variations) and φ (mean phase value) are calculated using data within the highlighted regions, given in Supplementary Table 3.

Sample ID	Sample description	Hotplate bake	Topography	Phase		Phase difference
			R_q (nm)	φ (°)	r_{sp}	$\Delta\varphi$ (°)
S1	PAGE (1LG region)	–	0.15	50.5	0.1	- 7.8
	PAGE (PVA region)	–	0.79	42.7		
S2	As-exfoliated (conventional)	√	0.12	1.3	1	5.7
	After PVA coating and water rinse	√	0.21	7	1	
S3	As-exfoliated (conventional)	√	0.14	1	1	5
	After PVA coating and water rinse	√	0.18	6	1	
S4	As-exfoliated (conventional)	√	0.16	1.1	1	3.4
	After PVA coating and water rinse	√	0.17	4.5	1	
S5	As-exfoliated (conventional)	√	0.13	3.4	1	6.2
	After PVA coating and water rinse	√	0.16	9.2	1	
S6	As-exfoliated (conventional)	√	0.14	1.5	1	2.1
	After PVA coating and water rinse	√	0.15	3.6	1	
S7	As-exfoliated (conventional)	√	0.176	4.2	1	11
	After water rinse	√	0.19	15.2	0.7	
S8	As-exfoliated (conventional)	√	0.181	6.1	1	29
	After water rinse	√	0.193	35.1	0.7	

Supplementary Table 3: Summary of surface rms roughness (R_q) and the mean phase value (φ) from the AFM topography and phase images for the different samples in Supplementary Fig. 13 – 20. The phase difference was calculated as $\Delta\varphi = \varphi$(after PVA and/or water exposure) – φ(as-exfoliated). The phase difference for S1 was calculated as $\Delta\varphi = \varphi$(PVA region) – φ(graphene region). Note that the r_{sp} value was adjusted during the AFM measurements in order to obtain good quality images.

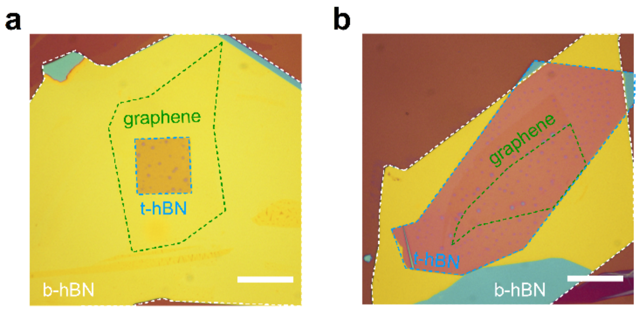

Supplementary Fig. 21: (a) Optical image of a stack prepared by our technique after the lamination step. (b) Optical image of a BGB stack prepared using the polymer-free layer assembly. In these images, the blue dashed line indicates the top hBN region; the green dashed line indicates the monolayer graphene region; the white dashed line indicates the bottom hBN region). Scale bars are 20 μm.

Supplementary References:

[1] Hwang, E.H. *et al.* Acoustic phonon scattering limited carrier mobility in two-dimensional extrinsic graphene. *Physical Review B* **77**, 115449 (2008)

[2] Park, C.H. *et al.* Electron-Phonon Interactions and the Intrinsic Electrical Resistivity of Graphene. *Nano Letters* **14**, 1113-1119 (2014)

[3] Purdie, D.G. *et al.* Cleaning interfaces in layered materials heterosturectures. *Nature Communications* **9**, 5387 (2018)

[4] Komatsu, K. *et al.* Observation of the quantum valley Hall state in ballistic graphene superlattices. *Science Advances* **4**, eaaq0194 (2018)

[5] Magonov, S.N. *et al.* Phase imaging and stiffness in tapping-mode atomic force microscopy. *Surface Science* **375**, L385-L391 (1997)
