# Supplementary material for: Versatile construction of van der Waals heterostructures using a dual-function polymeric film
Source: Nat Commun. 2020 Jun 15;11:3029. doi: 10.1038/s41467-020-16817-1 (PMC7295972; doi:10.1038/s41467-020-16817-1)
Supplement: Supplementary file 2 — Description of Additional Supplementary Files [file 41467_2020_16817_MOESM2_ESM.pdf]

Description of Additional Supplementary Files

File Name: Supplementary Movie 1

Description: The movie shows an example of our layer transfer process, in which a monolayer graphene residing on a PVA-coated substrate was picked up using a PPC/PDMS stamp.
